# Pharmacological Evaluation of Signals of Disproportionality Reporting Related to Adverse Reactions to Antiepileptic Cannabidiol in VigiBase

**DOI:** 10.3390/ph16101420

**Published:** 2023-10-05

**Authors:** Fabrizio Calapai, Carmen Mannucci, Liana McQuain, Francesco Salvo

**Affiliations:** 1 Department of Chemical, Biological, Pharmaceutical and Environmental Sciences, University of Messina, 98125 Messina, Italy; fabrizio.calapai@unime.it; 2 Department of Biomedical and Dental Sciences and Morphological and Functional Imaging, University of Messina, 98125 Messina, Italy; 3 Université de Bordeaux, European Training Programme in Pharmacovigilance and Pharmacoepidemiology (Eu2P), F-33000 Bordeaux, France; liana.mcquain@gmail.com; 4 CHU de Bordeaux, Service de Pharmacologie Médicale, Centre Régional de Pharmacovigilance de Bordeaux, F-33000 Bordeaux, France; francesco.salvo@u-bordeaux.fr; 5 Université de Bordeaux, INSERM, Bordeaux Population Health, U1219, AHeaD Team, F-33000 Bordeaux, France

**Keywords:** cannabidiol, cannabis, adverse reaction, adverse event, pharmacovigilance, serotonin, 5-HT_1A_ receptor

## Abstract

Cannabidiol is the first cannabis-derived drug approved for the treatment of Lennox–Gastaut syndrome, Dravet syndrome, and Tuberous Sclerosis Complex. In the current study, we performed a descriptive analysis followed by a disproportionality analysis of potential adverse events caused by CBD extracted from the VigiBase^®^ database. Furthermore, the biological plausibility of the association between CBD and the serotonin 5-HT_1A_ receptor as a possible cause of adverse events was analyzed and discussed. Data were extracted from the VigiBase^®^ database using the VigiLyze^®^ signal detection and signal management tool. Adverse events in VigiBase^®^ reports were coded using MedDRA, version 19 of Preferred Terms (PTs). Data were uploaded into SPSS software and analyzed via a disproportionality analysis. Statistically significant disproportionality signals for CBD were found for “weight decreased” (5.19 (95% CI: 4.54–5.70)), “hypophagia” (3.68 (95% CI: 3.22–5.27)), and “insomnia” (1.6 (95% CI: 1.40–1.83)). Positive IC025 values were found for “weight decreased” (2.2), “hypophagia” (1.3), and “insomnia” (0.5), indicating a surplus of reported cases. CBD’s interactions with 5-HT_1A_ serotonin receptors may offer a potential biological explanation for the occurrence of insomnia in patients. It is noteworthy that the risk profiles mentioned in the information for prescribing CBD as an antiepileptic agent by regulatory agencies showed disparities specifically related to the adverse event “insomnia”.

## 1. Introduction

*Cannabis sativa* is a plant containing more than 100 active cannabinoids, and several of them have been identified and isolated [1]. Cannabinoids are a group of chemicals having a characteristic C21 terpenophenolic backbone. They have been used for various purposes throughout history, including religious, recreational, and medicinal uses, specifically due to their anti-inflammatory, antiemetic, and anxiolytic effects, and they have also been used to treat seizure disorders [2,3]. Among the many cannabinoids present in *Cannabis*, two of the most abundant and well-known ones are the substances delta-9-tetrahydrocannabinol (THC) and cannabidiol (CBD). THC and CBD, indeed, have the same molecular formula, C_21_H_30_O_2_, but their structures differ slightly, resulting in different biological properties and pharmacological activities. These structural differences result in variations in how THC and CBD interact with brain receptors (e.g., cannabinoid receptors) and other biological targets, leading to distinct pharmacological activities and effects between the two compounds. In particular, CBD was isolated from the plant in the 1940s and, initially, it was thought to be non-active. Later, however, CBD was recognized to have different biological and pharmacological activities compared to THC, caused by its own pharmacokinetic and pharmacodynamic profile. Other major compounds were found in the *Cannabis sativa* plant, such as tetrahydrocannabivarin, cannabinol, cannabigerol, and cannabichromene [4].

Chronic *Cannabis* use is likely to lead to positive effects such as analgesic, anti-emetic, and antianorexic effects, improving one’s appetite. Recreational *Cannabis* use is known to cause a “high” or intoxicating effect, primarily due to THC’s psychotomimetic properties. CBD does not cause THC-like euphoric and psychotomimetic effects and has garnered increasing attention and popularity due to its potential therapeutic benefits. While THC and CBD are both present in *Cannabis*, THC has generally been found in higher concentrations compared to CBD [5]. However, the exact ratios of THC to CBD can vary depending on the specific *Cannabis* strain or cultivar. These differences in cannabinoid composition contribute to the varying effects and uses of different *Cannabis-*derived products [6].

The pharmacological activity and therapeutic applications of THC, the active component of *Cannabis*, have been prominently studied, particularly in recent years. THC activates the cannabinoid receptor 1 (CB1), principally expressed in the central nervous system (CNS), and cannabinoid receptor 2 (CB2), principally expressed in immune tissues [7,8]. The exact mechanisms of CBD are not yet fully understood, even though significant progress has been made through randomized clinical trials, the use of animal model studies, and the analysis of real-world data [9,10,11]. CBD was shown to have a weak link with the CB1 receptor and the CB2 receptor [12], but it binds to the peroxisome proliferator-activated receptor gamma [13] and has a high affinity for the orphan receptor GPR55 [14]. Some of the cannabinoid-mediated effects attributed to CBD may be due to its ability to inhibit endocannabinoid degradation due to the FAAH enzyme, thus increasing the level of anandamide [15]. CBD has been found to be the most potent and efficacious phytocannabinoid, activating the transient receptor potential vanilloid (TRPV)2 and, at lower values, TRPV1 as well [16]. CBD has the capability to inhibit the reuptake of the nucleoside adenosine, prolonging the activity of this endogenous neurotransmitter [17]. The equilibrative nucleoside transporter type (ENT)-1, regulating the concentration of adenosine, is inhibited by CBD, causing indirect agonistic activity of the adenosine receptor signaling [18]. CBD is an allosteric modulator in the mu and delta opioid receptors [19] and also has inhibitory activity on sodium, calcium, and potassium channels, which partially explains its anticonvulsant effects [20]. CBD exhibits agonistic activity on the serotonin 5-HT_1A_ receptors. In this regard, it is noteworthy that CBD is a full agonist towards the serotonergic 5-HT_1A_ receptor. Like serotonin, CBD increases [35S] GTPcS binding at this receptor and reduces the cAMP concentration at equivalent levels of receptor occupancy [21]. The [35S] GTPgammaS assay measures the level of G protein activation following occupation by the agonist [22]. The neurotransmitter serotonin is believed to be involved in several effects induced by cannabis, such as the relief of anxiety and pain [23], and it is also of great importance in hedonic tone, the reinforcement processes [24], and the sleep–wake cycle [25]. Furthermore, CBD has been proven to directly activate the serotonin 5-HT_1A_ receptors, which are associated with mood regulation, anxiety, and stress responses, suggesting that this compound can reduce acute autonomic responses to stress and their delayed emotional consequences by facilitating 5-HT_1A_ receptor-mediated neurotransmission [26].

The use of CBD is generally considered safe; however, its safety profile has not been completely defined. CBD is authorized for medicinal use as “add-on” therapy for seizures in patients 2 years of age and older affected by Lennox–Gastaut syndrome or Dravet syndrome in association with clobazam. Furthermore, it has been licensed as adjunctive treatment of seizures in patients 2 years of age and older affected by tuberous sclerosis complex. The list of important risks for CBD licensed as antiepileptic agent includes hepatocellular injury, somnolence and sedation, lethargy, pneumonia, and rash hypersensitivity reactions [27]. This information is based on available data obtained through clinical studies, which have supported the requirement of medicinal authorization for products based on CBD. The existing real-world data are poor, even though, with a recent previous study analyzing the European database EudraVigilance, we suggested that precautions should be adopted for the appropriate monitoring of CBD’s potential adverse effects when it is used as antiepileptic agent. Such precautions include an awareness of interactions with other drugs, the aggravation of epilepsy, and drug effectiveness in reducing epilepsy [28]. Starting from this point of view, in the present study, the relationship between CBD’s safety profile and serotonin’s involvement in potential adverse reactions has been investigated. In particular, the objectives of the study were to perform a descriptive analysis of data from spontaneous reports of adverse reactions to CBD licensed by drug regulatory agencies as an antiepileptic agent in the VigiBase^®^ database, followed by a disproportionality analysis of data on selected adverse events potentially associated with the activity of CBD at the serotonin 5-HT_1A_ receptor level, and finally to investigate the biological plausibility of the interaction between this receptor and CBD.

## 2. Results

### 2.1. Characteristics of the Reports

The reports examined in this study were sent from various countries, with the United States of America (USA) being the most represented; the other countries whose reports we analyzed were France, Spain, Italy, Canada, Australia, Uruguay, and the United Kingdom. From January 2009 to 15 May 2023, the database VigiBase^®^ collected 12,702 ICSRs related to CBD use, of which 5466 (43.0%) were corresponding to recognized criteria for serious adverse reactions.

A total of 820 ICSRs (6.5% of all CBD-related ICSRs) correspond to events potentially linked to 5-HT_1A_ receptor activation. These mainly originated from the USA (754; 92.0%) and mostly from consumers (463 reports; 56.5%). Gender was frequently not reported (524; 63.9%), while in the remaining portion there was a substantial balance between data from men and women (149 vs. 147, respectively). Stratification for age suggests that the age group most affected is represented by adults aged between 18 and 44 years, with 57 cases (7%), even if in most of the cases the age of the subject was not reported (685; 83.5%). The most used product was Epidiolex (753 ICSRs, 91.8%), while products in the other cases were distributed as follows: CBD oil, 15 cases (1.8%); unknown CBD formula, 47 (5.7%). The most represented diseases for which CBD was employed were Lennox–Gastaut Syndrome (315 reports; 38.4%) and epilepsy (200 reports; 24.4%; Table 1).

### 2.2. Disproportionality Analysis

Among the selected events, “Weight decreased” was related to the highest ROR values with 5.2 (95% CI 4.5; 5.7), followed by “Hypophagia” 3.7 (3.2; 5.3), and “Insomnia” 1.6 (1.4; 1.8). “Dizziness” and “Palpitations” were related to a negative ROR (0.2 [0.2–0.3] and 0.1 [0.1; 0.2], respectively).

IC component results confirmed the ROR ones. Data on IC025, extracted from the VigiBase^®^ database, were also reported. These data show an IC positive for the following adverse reactions: “Weight decreased”, “Hypophagia”, and “Insomnia” (Table 2).

### 2.3. Characteristics of Insomnia Reports

Among the signals of the adverse reactions analyzed, only “Insomnia” is not mentioned in other previous studies nor within the summary of product characteristics (SmPC) of Epidiolex, a medicinal product regularly licensed with authorization for entry in the drug market and based on CBD [29]. Thus, we performed a post hoc analysis only on its related ICSRs (*n* = 221). As already seen for the general data, here too there are not many ICSRs that report data on age (25; 11.3%), but among those in which this information is present, the most represented age group is that of children from 2 to 11 years (8; 3.6%). As for sex, we also find in this case a slightly more representative number for male than female patients (28 vs. 25; 12.7% and 11.3%). Regarding the drugs involved, the use of the medicinal product Epidiolex (206; 93.2%) stands out (Table 3).

**Figure 1:**
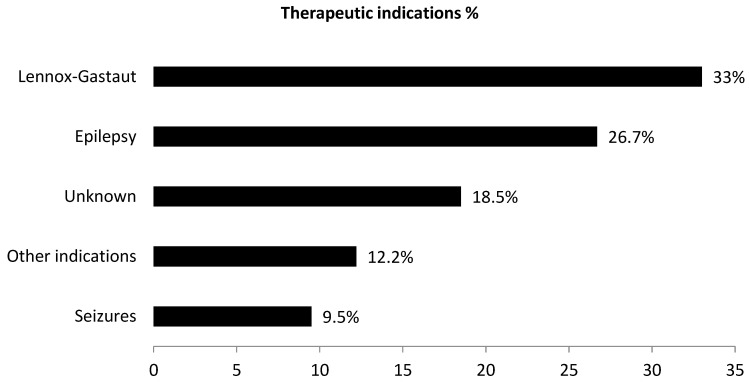
Therapeutic indications of individual cases of insomnia (*N* = 221) as suspected adverse reaction to cannabidiol.

For the adverse reaction “Insomnia”, the most represented therapeutic indications are Lennox–Gastaut and epilepsy, with 73 (33.0%) and 59 (26.7%) reports, respectively (Figure 1). In the cases of insomnia found in individual reports, the number of serious cases reported was 48 (21.7%).

## 3. Discussion

Quantitative analysis of the spontaneous adverse drug reaction signaling is a routine activity in research defining drug safety and the reporting odds ratio (ROR) and information component (IC) are among the most common methods used. The results of the present study show that adverse reactions linked to CBD, which are traceable in the database VigiBase^®^, are mostly reported when people take the licensed medicinal product Epidiolex, generally used as antiepileptic agent, while the adverse reactions linked to unlicensed CBD oil are reported in a minor percentage. The application of statistical methods reveals that among all the adverse events linked to CBD potentially associated with the serotonin receptor 5HT_1A_, the ROR is increased for “Weight decreased”, “Hypophagia”, and “Insomnia”. Both “Weight decreased” and “Hypophagia” are adverse reactions included in the risk management information published for CBD use as drug; however, “Insomnia” has not been previously signaled as an adverse reaction according to the data available from the clinical studies carried out on the antiepileptic effects of CBD.

The ROR indicates the odds of an adverse reaction occurring with the use of a substance or a drug in comparison to the odds of the same kind of adverse reaction occurring with all the other substances or drugs traceable in the database [30]. The information component (IC) indicates whether a particular association between a substance or a drug and an adverse reaction is signaled more often than can be expected in any of the remaining adverse reactions collected in the database. In this case, the value of IC is positive, and it means that the event/drug association is not expected. When the substance (or drug) and the particular adverse reaction are not dependent, the value of IC results to be zero [31]. In the present study, the calculation of ROR for the adverse reaction “Insomnia” reveals that the risk linked to its occurrence is moderately high (1.60; C.I. 1.40–1.83), and the IC analysis indicates that it is signaled more often than expected. Insomnia is a sleep disorder defined as chronic dissatisfaction with sleep quantity and/or quality. It is associated with difficulty initiating and/or maintaining sleep, early morning awakening, and unrefreshing sleep [32].

Despite the increased use of medicinal cannabis to treat insomnia and other sleep disorders, the evidence supporting the therapeutic utility of cannabinoid therapies in sleep disorders is not strong [33]. While positive effects of CBD against insomnia were observed in an old small clinical study, more recent clinical research has shown that sleep architecture polysomnography was not changed through the acute administration of 300 mg of CBD [34]. CBD is generally considered a substance causing somnolence rather than insomnia, even though the effects of CBD on sleep are not yet fully understood. In clinical studies in which 25/mg/kg of CBD was given daily to children affected by epilepsy, somnolence and sedation were detected. However, CBD reduces the metabolism of anticonvulsants when they are concomitantly used, and thus, the sedation observed in these clinical trials could be indirectly caused by CBD’s inhibitory effect [35]. Drowsiness was also reported as one of the most common side effects in a Phase 1 clinical trial of CBD carried out in healthy volunteers but without any difference compared to placebo, and the highest frequency of the effect was detected with the much higher dose of 6000 mg, while the unit dose of CBD as an antiepileptic is 100 mg [36]. Furthermore, insomnia related to CBD use could be due to the CBD’s known activity as a negative allosteric modulator of the CB1 receptor [37], so the possibility exists that CBD could exhibit either stimulating or inhibiting properties at different doses.

The biological plausibility of the link between CBD and insomnia has to be found in the mechanism of action of the cannabinoid. As mentioned above, CBD can influence the serotonergic system activity, in particular through its action on the 5-HT_1A_ receptor. Serotonergic neurons in the brain stem raphe nuclei are responsible for the initiation and maintenance of slow-wave sleep and for the ‘priming’ of rapid eye movement sleep [38]. 5-HT_1A_ autoreceptors play a role in controlling the serotonergic tone through negative feedback inhibition in response to increases in the neurotransmitter serotonin; thus, the growing autoreceptor desensitization may be the cause for the delayed onset of action of the selective serotonin reuptake inhibitors (SSRIs) used as antidepressants [39]. The 5-HT_1A_ receptor subtype is a heptahelical G protein-coupled receptor associated with inhibitory G proteins (Gi/Go) [40] and is widely distributed throughout the central nervous system (CNS) in both pre- and postsynaptic sites. It is one of the most abundant and widely expressed receptors in the brain, both as an autoreceptor or a heteroreceptor [41]. The 5-HT_1A_ autoreceptor plays a role in controlling the serotonergic tone through negative feedback inhibition in response to increases in serotonin; thus, the growing autoreceptor desensitization may be the cause for the delayed onset of action of SSRIs. The expression of the 5-HT_1A_ receptor in the limbic system and brain stem raphe nuclei supports its role in regulating functions like mood and memory [42,43]. In this regard, it has been hypothesized that the activation of the 5-HT_1A_ receptor modulates anxiety [44] and the response to antidepressant drugs [45].

It has been shown that the systemic administration of 8-hydroxy-2-(di-*n*-propilamino)tetralin (8-OH-DPAT), a selective 5-HT_1A_ receptor agonist [46], in doses ranging from 0.010 to 0.375 mg/kg consistently increases waking and reduces slow-wave sleep and rapid eye movement sleep [47]. Finally, the role played by serotonin, and in particular of its activity at the 5-HT_1A_ receptor, in the regulation of the sleep/wakefulness cycle is controversial. It is thought that serotonergic agonistic activity towards the 5-HT_1A_ receptors could increase waking or sleep depending on the route of administration and the brain region involved [48]. Adverse reactions reported through spontaneous signals extracted from another database, the European EudraVigilance database, collecting spontaneous signaling reports of adverse reactions have been partially described in a previous article. Based on a descriptive analysis, that article suggested adopting appropriate monitoring of adverse reactions potentially caused by CBD, together with more caution about its use as an antiepileptic, with particular attention to pharmacological interactions, the aggravation of epilepsy, and the lack of drug effectiveness [28]. The present analysis shows important differences: first of all, this study used VigiBase^®^, a database including spontaneous reports of adverse reactions sent also by extra-European countries, and secondly and more importantly, this study applied a disproportionality analysis. With respect to previous clinical research or other analyses conducted investigating CBD safety, from the analysis of the data from VigiBase^®^ emerges a potential risk of occurrence of insomnia associated with therapy using this substance. This is a new important issue regarding CBD safety, requiring more in-depth information and training for physicians and consumers, especially in the light of its growing availability without prescription [49].

## 4. Material and Methods

Data were extracted from the Uppsala Monitoring Centre (UMC) database VigiBase^®^. VigiBase^®^ holds over 30 million anonymized spontaneous individual case safety reports (ICSRs) of suspected adverse effects related to the use of medicines (as of January 2023), received from 130 country members since 1967 [50].

In this database, the information is recorded in a structured form. Its purpose is to provide evidence from which potential medicine safety hazards (signals) may be detected and successively communicated. Demographic characteristics (age, sex, area of residence, notifier’s country) and details concerning the reported effect (suspected drugs, concomitant drugs, adverse drug reaction, date of occurrence, and seriousness) are collected in the database [51]. Adverse events in Vigibase^®^ reports are coded using the Medical Dictionary for Regulatory Activities (MedDRA, version 19.0) of Preferred Terms (PTs). PTs of MedDRA are intended to represent a single medical concept, linked with broader Higher-Level Terms (HLT), Higher-Level Group Terms, and System Organ Classes (MedDRA Hierarchy, 2023) [52]. All cases linked to CBD administration recorded in Vigibase^®^ up to 15 May 2023 were analyzed.

### 4.1. Case of Interest Definition

We sought a potential pharmacovigilance signal for the use of medical CBD and adverse reactions potentially associated with the serotonin receptor 5HT_1A_ using the following PT terms: weight decreased, insomnia, dizziness, hypophagia, and palpitations.

### 4.2. Exposure Definition

All reports of the term “Cannabidiol” recorded in Vigibase^®^ up to 15 May 2023 were identified using the Vigibase browser Vigilyze^®^.

### 4.3. Statistical Analysis

A descriptive analysis was presented, in the form of frequencies and percentages, for adverse reactions potentially linked to CBD use as an antiepileptic agent contained in the database VigiBase^®^ until December 2022. All statistical analyses were completed using SPSS statistical software, version 28.0 (SPSS, IBM, Armonk, NY, USA).

A disproportionality analysis by using data from VigiBase^®^, the World Health Organization’s Pharmacovigilance Database, was performed based on two different measures, the reporting odds ratio (ROR) and the information component (IC). The ROR, as an approximation of the odds ratio (used in case-control studies), is used to assess the strength of disproportionality. Reports linked to CBD were compared with all the other ICSRs in VigiBase^®^. An ROR equal to 1 states the absence of a signal; conversely, an ROR greater than 1 indicates a signal and the existence of an association. The higher the ROR, the stronger the association. The precision of the approximate ROR is reflected by a 95% confidence interval (95% CI). Consequently, an ROR is statistically significant when the lower bound of its 95% CI is greater than 1 [53,54]. The IC compares the observed and the expected values for the combination of a given drug and an adverse drug reaction (ADR) to yield associations between them. The positivity of the IC reveals the superiority of the number of observed reports over the number of expected reports. The IC is a logarithmic measure of the strength of the association between a drug and a single type of adverse reaction [55].

We followed as a guide for our research “The **Re**porting of **A D**isproportionality analysis for dr**U**g **S**afety signal detection using spontaneously reported adverse events in **P**harmaco**v**igilance (READUS-PV)”. READUS-PV is an international collaborative that aims to develop the first reporting recommendations for studies using disproportionality analyses in databases of spontaneously reported adverse events accessed on 1st of August 2023 (https://readus-statement.org).

## 5. Conclusions

In conclusion, analysis of real-world data from VigiBase^®^ suggests that, in terms of risks linked to the use of medical CBD, insomnia could emerge as potential adverse reaction not signaled by clinical trials conducted before registration and, consequently, not considered in the risk management of licensed drugs containing it. The biological plausibility of a causal relationship between CBD and insomnia can be explained through the current knowledge of the mechanism of action of the cannabinoid, including its potential influence on serotonergic system activity by acting on the 5-HT_1A_ receptor. However, in order to reach definitive or more stable conclusions, better-quality data are surely needed. Further in-depth studies will provide clearer results that can be communicated to physicians and consumers prescribing or using CBD for medical use or for well-being. In addition, as people who suffer from insomnia caused by cannabidiol seem to be mostly patients with epilepsy, it is important to verify the external validity of these results, with the aim to determine the extent to which it is possible to generalize the findings of this study to the general population.

## Figures and Tables

**Table 1:** Main characteristics of individual case safety reports (ICSRs) related to cannabidiol (CBD) collected in VigiBase^®^.

Main Characteristics	N° Cases (820)	%
	Patient Age	
28 days–23 months	1	0.1%
2–11 years	23	2.8%
12–17 years	23	2.8%
18–44 years	57	7.0%
45–64 years	18	2.2%
65–74 years	6	0.7%
≥75 years	6	0.7%
Unknown	685	83.5%
28 days–23 months	1	0.1%
	**Sex**	
Male	149	18.2%
Female	147	17.9%
Unknown	524	63.9%
	**Countries**	
USA	754	92.0%
UK	18	2.2%
France	12	1.5%
Germany	8	1.0%
other countries	28	3.4%
	**Reporter qualification**	
Physician	154	18.8%
Pharmacist	22	2.7%
Other Health Professional	179	21.8%
Consumer/Non Health Professional	463	56.5%
Unknown	8	1.0%
	**Drug/Product**	
Epidiolex	753	91.8%
Unknown (Cannabidiol)	47	5.7%
CBD oil	15	1.8%
Convupidiol (Argentina)	3	0.4%
Xannadiol (Uruguay)	2	0.2%
	**Indication**	
Epilepsy NOS	200	24.4%
Lennox Gastaut Syndrome	315	38.4%
Seizures	40	4.9%
Partial seizure	41	5%
Idiopatic epilepsy	34	4.1%
Tuberous Sclerosis Complex	11	1.3%
Product use for unknown indication	79	9.6%
Other indications	100	12.2%
	Serious/non serious adverse reactions	
Yes	233	28.4%
No	587	71.6%

**Table 2:** Reporting odds ratio (ROR), information component (IC) and IC025 of individual cases of suspected adverse reactions to cannabidiol (CBD) reported in VigiBase^®^ signaling as suspected adverse reactions “weight decreased”, “hypophagya”, “insomnia”, “dizziness”, “palpitations”.

Adverse Reaction	N. of Cases	ROR (95% CI)	IC (IC025)
Weight decreased	456	5.19° (4.54–5.70)	2.4 * (2.2)
Hypophagia	30	3.68° (3.22–5.27)	1.8 * (1.3)
Insomnia	221	1.60° (1.40–1.83)	0.7 * (0.5)
Dizziness	118	0.23 (0.20–0.27)	−2.1 (−2.4)
Palpitations	16	0.12 (0.10–0.19)	−3.1 (−3.9)

° = statistically significant when the lower bound of 95% CI is greater than 1. * = positivity of the IC reveals the superiority of the number of observed reports over the number of expected reports.

**Table 3:** Main characteristics of individual case safety reports (ICSRs) of insomnia related to cannabidiol (CBD) collected in VigiBase^®^.

Main Characteristics	N° Cases (221)	%
	**Patient Age**	
2–11 years	8	3.6%
12–17 years	5	2.3%
18–44 years	4	1.8%
45–64 years	4	1.8%
65–74 years	2	0.9%
≥75 years	2	0.9%
Unknown	196	88.7%
	**Sex**	
Male	28	12.7%
Female	25	11.3%
Unknown	168	76.0%
	**Drug/Product**	
Epidiolex	206	93.2%
Unknown Cannabidiol	11	5.0%
CBD oil	2	0.9%
Convupidiol (Argentina)	2	0.9%

## Data Availability

The data presented in this study are contained within the article. Members of the public can access a limited view of VigiBase^®^ data, via WHO’s VigiAccess website.

## Abbreviations

CBD	cannabidiol
CB1	cannabinoid receptors 1
CB2	cannabinoid receptor 2
CI	confidence interval
ENT-1	equilibrative nucleoside transporter type 1
FAAH	fatty-acid amide hydrolase 1
GPR55	G protein-coupled receptor 55
HLT	Higher-Level Terms
8-OH-DPAT	8-hydroxy-2-(di-*n*-propilamino)tetralin
IC	information component
ICSRs	individual case safety report
MedDRA	Medical Dictionary for Regulatory Activities
PTs	Preferred Terms
READUS-PV	Reporting of A Disproportionality analysis for drUg Safety signal detection using spontaneously reported adverse events in Pharmacovigilance
ROR	Reporting odds ratio
SSRIs	selective serotonin reuptake inhibitors
THC	delta-9-tetrahydrocannabinol
TRPV	transient receptor potential vanilloid
UMC	Uppsala Monitoring Centre
